# Life in a fishbowl: Space and environmental enrichment affect behaviour of *Betta splendens*

**DOI:** 10.1017/awf.2024.1

**Published:** 2024-01-16

**Authors:** Ronald G Oldfield, Emily K Murphy

**Affiliations:** Department of Biology, Case Western Reserve University, 10900 Euclid Ave, Cleveland, OH 44106, USA

**Keywords:** aggression, animal welfare, aquarium, companion animal, fish, pet

## Abstract

The public has expressed growing concern for the well-being of fishes, including popular pet species such as the Siamese fighting fish (*Betta splendens*). In captivity, male *Bettas* behave aggressively, often causing injuries and death if housed together. As a result, they are typically isolated in small fishbowls, which has been widely criticised as cruel. To investigate the impact of keeping *Bettas* in these conditions, we recorded the behaviour of individual males in containers of different sizes that were either bare or enriched with gravel, large rocks, and live plants. When male *Bettas* were housed individually in small bowls (0.5 L) they spent less time swimming than they did when they were kept in larger aquaria (10, 38, and 208 L). Fish that were kept in enriched containers exhibited more instances of swimming. To determine if two male *Bettas* housed together might coexist peacefully if given enough space and cover from plants and large rocks, we quantified the behaviour of pairs of male *Bettas* in bare or enriched aquaria of different sizes (10, 38, 208, 378 L). Fish performed fewer approaches and aggressive displays, but not attacks, and more bouts of foraging, when in larger aquaria. This study shows that the small fishbowls typically used in pet stores suppress swimming behaviour in male *Bettas* and at least a 10-L aquarium is required to ensure full expression of swimming behaviour. Furthermore, even the use of very large aquaria cannot guarantee peaceful cohabitation between two males.

## Introduction

The public has expressed growing concern for how humans treat animals of other species, including fishes. The first research on the welfare of fishes was stimulated by public concern over the conditions faced by food fishes on fish farms in Europe (Kristiansen & Bracke 2020). Concern has since spread to address fish welfare in other contexts such as biomedical research and commercial and recreational fishing (for reviews, see Huntingford *et al.* 2006; Branson 2008; Kristiansen *et al.* 2020). Concern over animal welfare goes beyond physical health and includes consideration of an animal’s psychological and social well-being (Veasey 2017). Studies on well-being of fishes often investigate aspects of confinement such as space limitation, environmental complexity, and social group composition (Oldfield & Bonano 2023), and they are typically conducted by observing and quantifying behaviour (Martins *et al.* 2012; Watters *et al.* 2021). They seek not only to eliminate negative conditions and experiences, but also to elicit positive experiences (Boissy *et al.* 2007; Balcombe 2009). Despite the burgeoning of this new research on the welfare of fishes, ornamental fishes kept in home aquaria have been largely overlooked. In an aquarium, a fish may encounter conditions which may have harmful negative effects, or positive beneficial effects, on its well-being (Fife-Cook & Franks 2019). Several recent reviews have discussed the welfare of aquarium fishes (Walster *et al.* 2015; Stevens *et al.* 2017; Torgersen 2020; Brandao *et al.* 2021), but few studies have analysed their behaviour to assess their well-being (Saxby *et al.* 2010; Oldfield 2011; Sloman *et al.* 2011; Smith & Gray 2011).

**Figure 1.**
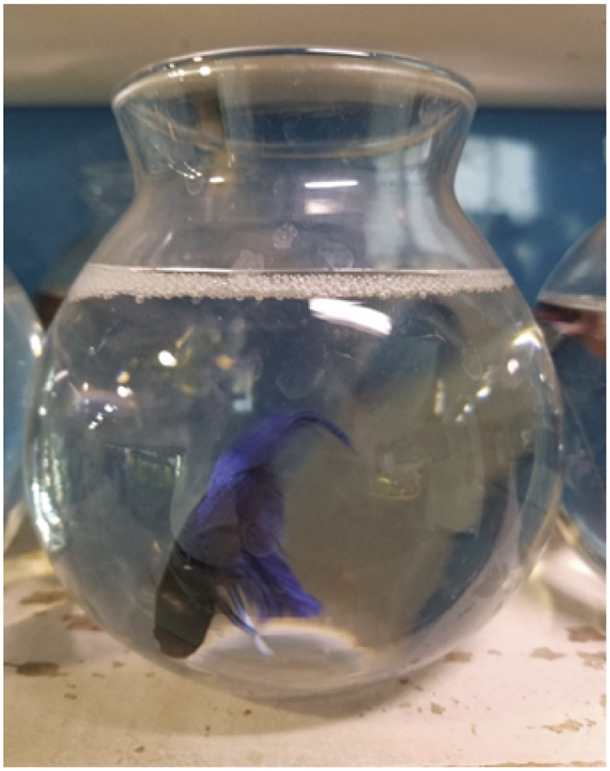
Photograph showing a male *Betta splendens* in a small (0.5-L) fishbowl at a retail pet store. Photograph courtesy of Ron Oldfield.

One ornamental fish species, the Siamese fighting fish (*Betta splendens*), is aggressive to conspecifics so pet stores keep individual males isolated in small fishbowls around 0.5 L in volume (Figure 1). Producers keep individuals in volumes even smaller, with researchers recommending 150 mL for permanent holding (Saekhow *et al.* 2018) and 80 mL for transport (Thongprajukaew *et al.* 2023). Organisations such as People for the Ethical Treatment of Animals (PETA) consider this practice to be cruel, and have campaigned to end the practice of keeping *Bettas* in small bowls (The Herald 2009; PETA 2021). *Betta splendens* is a tropical freshwater fish native to Thailand. Their natural habitat consists of shallow ponds and pools with thick plant cover and stagnant water, a condition they can tolerate due to their labyrinth organ, which allows them to breathe atmospheric air at the water’s surface. In the wild, male *Bettas* coexist by forming territories at a density of 1.7 individuals m^–2^ (Pleeging & Moons 2017). The fishbowls in which *Bettas* are held would seem likely to negatively affect a *Betta’*s well-being by preventing it from moving about. Most *Betta* care guides suggest a minimum volume of at least 9.5 to 11 L (e.g. Betta Fish Care Guide 2021; Stanton 2021). However, those recommendations are based purely on personal opinion — there is yet to be any research carried out on the well-being of *Bettas.* The effect of available space on locomotory behaviour in *Bettas* has not been investigated.

It is also not known whether captive male *Bettas* would peacefully coexist if provided with space and physical cover approximating their natural territories. Some studies have investigated social behaviour of *Bettas* held in groups in large aquaria. Goldstein (1975) observed social behaviour in *Bettas* held in a large L-shaped aquarium with 2-m long arms and a total volume > 850 L. In addition to the large volume of water available, the aquarium included rooted vegetation for physical enrichment. Under those conditions, Goldstein established a stable community of *Bettas* that included both males and females, but aggression resulted in the death of several fish prior to establishment of a stable community. Cain *et al.* (1980) specifically tested the effect of space on aggressive behaviour in male *Bettas.* They placed pairs of males into three differently sized aquaria: small (3.8 L), medium (28.5 L) and large (75.7 L), and found that neither latency to first display, number of displays, nor duration of displays differed as a function of aquarium size, but the number of attacks was significantly lower in larger aquaria. They concluded that attacks are energetically more expensive to perform and not economically advantageous in larger aquaria where there is an increased probability of escape. Haller and Wittenberger (1988) and Haller (1994) kept multiple male *Bettas* together for up to seven days. They observed the formation of stable dominance hierarchies and quantified differences in metabolic costs between dominant and subordinate individuals. None of these studies provided fish with territorial densities approximating that found in nature.

In the current study, we set out to determine the minimum amount of space and environmental enrichment required for a lone male *Betta* to express unreduced rates of swimming behaviour and the amount of space and enrichment required for two male *Bettas* to coexist peacefully. To this end, two experiments were conducted to determine the effects of space and enrichment on locomotory behaviour and social behaviour. In Experiment 1, we quantified swimming behaviour performed by lone *Bettas* in small fishbowls and aquaria of various sizes, with and without environmental enrichment, with the expectation that individuals would perform less swimming behaviour in small, unenriched containers. In Experiment 2, we quantified aggressive behaviour in pairs of male *Bettas* placed in unenriched and enriched aquaria of various sizes. We expected aggression to be lowest in large, enriched aquaria.

## Materials and methods

### Study animals

Male *Bettas* were purchased from a retail pet store in Cleveland, Ohio and maintained at Case Western Reserve University in Cleveland, Ohio. The fish were exposed to natural sunlight and the photoperiod of Cleveland, supplemented with standard fluorescent ceiling lights. The amount of time exposed to artificial lighting varied by day. The temperature was kept between 21 and 22°C and fish were fed once daily with either commercial flake food or frozen food (*Daphnia* or *Cyclops* spp.).

### Experiment 1

In Experiment 1, eight male *Bettas* were each placed individually in a container of one of four sizes: a round, glass 0.5-L fish bowl (5-cm radius), a 10-L glass aquarium (31 × 21 × 16 cm; length × width × height), a 38-L glass aquarium (51 × 20 × 31 cm), or a 208-L glass aquarium (122 × 33 × 53 cm). Each container size was presented as both an unenriched and an enriched treatment, i.e. there were eight containers set up at any given time, and each trial involved one fish in each of the eight treatments. Unenriched treatments contained only water while enriched treatments included a layer of natural gravel, live unrooted plants (*Ceratophyllum demersum*), and one large rock that was approximately three-quarters the height of the container and placed in the middle of the container. Water filters were not used. Prior to the commencement of the trials, the fish were moved among the eight containers multiple times with each fish experiencing both an unenriched and an enriched environment at least once before data were collected.

To begin each trial, the water in a particular container was first replaced with aged tap water and then a fish was placed into that container, where it was habituated to the treatment overnight, visually isolated from other fish. The order that each fish experienced each treatment was random. Data collection then began at 1600h the following day. After all data had been collected on any given day, we either immediately moved all the fish to the next treatments (containing new aged tap water) so they could begin habituating for data collection the following day, or we left them in the experimental containers for 2–4 days and then moved them the day preceding the next scheduled day of data collection. This resulted in a habituation period that was consistently 22–26 h across all replicates. Each of the eight fish was observed in each treatment condition one time, once in each of the four unenriched treatments and once in each of the four enriched treatments, for a total of eight observations per fish. Fish were fed only after data recording was finished for the day.

Each fish was observed for 10 min in each treatment. During each 10-min period, each bout of swimming behaviour was recorded. A bout of swimming was defined as locomotory behaviour separated from other locomotory movements by at least one second. To sample all occurrences of swimming behaviour, we used the behaviour sampling rule and the continuous recording rule — each individual was observed continuously, and each observation was uninterrupted (Martin & Bateson 2007). To calculate time budget, we recorded the behaviour of each individual using the scan sampling rule and the instantaneous point sampling recording rule at 15-s intervals (Martin & Bateson 2007). These data were used to infer the amount of time each individual spent engaged in swimming behaviour.

To compare the number of swimming bouts performed across individuals, we constructed a Generalized Linear Mixed Model (GLMM) in SPSS 28 using Poisson distribution with log-link function (which fits count data in the form of positive integers). This procedure assumes that multiple records for a single subject represent repeated measurements. The data structure was set with ‘fish’ as subjects and ‘day’ as repeated measures. ‘Container size’, ‘enrichment’, and ‘container size × enrichment’ interaction were set as fixed factors. ‘Container size’ was set as a continuous, scale variable. ‘Subject’ was also included as a fixed factor to control for variation due to individual differences in behaviour. Model fit was assessed by size of Akaike Corrected Information Criterion and Bayesian Information Criterion values. The GLMM function in SPSS is not able to assess simple effects, so to determine which treatments significantly differed from one another, we performed two-way factorial analyses of variance using R version 4.0.3, with ‘swimming’ as the dependent variable, ‘container size’, ‘enrichment’, and ‘container size × enrichment’ interaction as independent variables, and Tukey’s Honest Significant Difference test to identify significant differences between treatments. We performed a similar procedure for time budget data, except in the GLMM we used a normal probability distribution with an identity link function because each data-point was a proportion of total time observed.

### Experiment 2

In Experiment 2, we used 16 male *Bettas.* These individuals differed from those used in Experiment 1, i.e. no fish was used in both Experiment 1 and 2. Each were housed alone in a 38-L glass aquarium (51 × 20 × 31 cm) that contained gravel and live unrooted plants (*Ceratophyllum demersum* and *Lemna minor*). Opaque barriers were placed between aquaria so that each *Betta* was visually isolated from other *Bettas* > 24 h prior to being subjected to a trial. We held the fish in these tanks for several weeks, and periodically ran preliminary trials in which they were removed and placed in one of the test tanks with another male (see below) to ensure that all fish had prior experience with the treatment conditions by the time trials began.

For each trial, two *Bettas* were netted from their holding tanks and placed into a glass aquarium of one of four sizes: 10 L (31 × 21 × 16 cm), 38 L (51 × 20 × 31 cm), 208 L (122 × 33 × 53 cm), and 378 L (183 × 46 × 51 cm). The two fish were placed, simultaneously, at opposite ends of the test tank. As in Experiment 1, each aquarium size was presented as both an unenriched and an enriched treatment, i.e. there was a total of eight test aquaria. Unenriched and enriched configurations were the same as in Experiment 1. As in Experiment 1, there were eight different treatments and eight replicates per treatment. However, because each replicate consisted of a pair of fish instead of an individual, fish were specifically chosen for each trial such that no two individuals were paired together more than once. We controlled for the repeated observations of the same individuals by using a repeated measures analysis that included the identity of each individual fish and the day of experimentation as factors in our statistical model (see below). Each pair was then randomly assigned to a particular combination of tank size and enrichment, and this resulted in some individuals experiencing the same treatment more than once. We controlled for prior experience in the same treatment conditions by ensuring that all subjects had prior experience in the various test tanks during preliminary trials (as described above).

Fish were observed for 10 min. All occurrences of behaviour and time budget were recorded as described for Experiment 1, except that we used an ethogram to capture a diversity of behaviour instead of focusing only on swimming (Table 1). Also, time budgets were quantified by recording behaviour of both fish at 30-s instead of 15-s intervals. To prevent injury, the two fish were separated if three bites occurred consecutively (three of 64 replicates had observation periods shorter than 10 min). At the end of each trial, fish were returned to their individual holding tanks and held for 1–3 days until their next trial.

**Table 1.** *Betta splendens* ethogram

Behaviour	Description
Rest	Motionless on substrate. Colouration often dark and fins not moving.
Hover	Motionless in water column.
Swim	Move through the water column without an obvious destination.
Approach	Swim towards another fish.
Display	Positioned near another fish and spread the median fins and/or flare the operculum and branchiostegal membrane.
Bite	Rapidly close the jaws on or near another fish.
Charge	Rapidly move toward another fish.
Chase	Rapidly follow another fish while it retreats.
Retreat	Move away from another fish in response to its movement.
Gulp air	Suck in air at the surface of the water.
Forage	Suck at gravel or engulf or chew floating particles.

To compare numbers of bouts of behaviour performed across treatments, we constructed GLMMs in SPSS 28 using Poisson distribution with log-link function as we did in Experiment 1. As before, ‘container size’ (called ‘aquarium size’ in Experiment 2), ‘enrichment’, ‘aquarium size × enrichment’ interaction, and ‘subject’ were set as fixed factors. Unlike in Experiment 1, in Experiment 2 individuals were not evenly distributed across treatments. Furthermore, *Bettas* tend to attack aggressive opponents more than non-aggressive ones (Cain *et al.* 1980). Therefore, ‘opponent’ was also included as a fixed factor in the models, i.e. each replicate pair was included twice in each analysis: once with one fish designated as the subject and the other fish designated as the opponent, and a second time with the roles reversed. To account for variation in the total minutes of each observation period, we used ln minutes as an offset variable in the model. The model was run one time for each behaviour pattern (Table 1). Outright physical attacks (‘bites’, ‘charges’, and ‘chases’) were scarce, so we summed the bouts of those behaviour patterns to form the new variable ‘total attacks’, which we then analysed using the GLMM. Again, we also performed two-way ANOVAs and Tukey’s Honest Significant Difference tests to determine the significant differences between treatments. We repeated these procedures for time budget data using a normal probability distribution with an identity link function and behaviour per minute as the dependent variable.

Finally, we compared across treatments the latency to perform the first bite. To quantify latency to first bite, each trial was observed for an extra 20 min, to result in a 30-min observation period. If a bite was performed within this period, then the identity of the fish performing the bite and the time at which it occurred were recorded. If no bite occurred during the 30-min period, then that trial was omitted from the data set. Out of the 64 total trials, aggression escalated to biting in 36 trials. We compared latency to first bite across treatments using a GLMM in SPSS 28 using a normal distribution with identity link function. Fixed factors were the same as in the analyses described previously, and latency to first bite was the target (dependent) variable.

### Ethical approval

Our experiments were approved by the Institutional Animal Care and Use Committee at Case Western Reserve University (# 2018-0092). Experiment 2 involved placing two individuals together and observing agonistic interactions. Before we ran any trials, we proactively established a procedure that required us to stop any interaction prior to it escalating to ongoing physical combat (i.e. a fight). In contrast to previous studies that allowed ongoing, repeated physical attacks between individuals (e.g. Goldstein 1975; Cain *et al.* 1980), we discontinued any trial by separating the two fish if three ‘bites’ occurred consecutively. No animals were harmed. At the end of the study, fish were either preserved to serve as teaching aids for an ichthyology course or were adopted as pets by students and staff.

## Results

### Experiment 1

**Figure 2.**
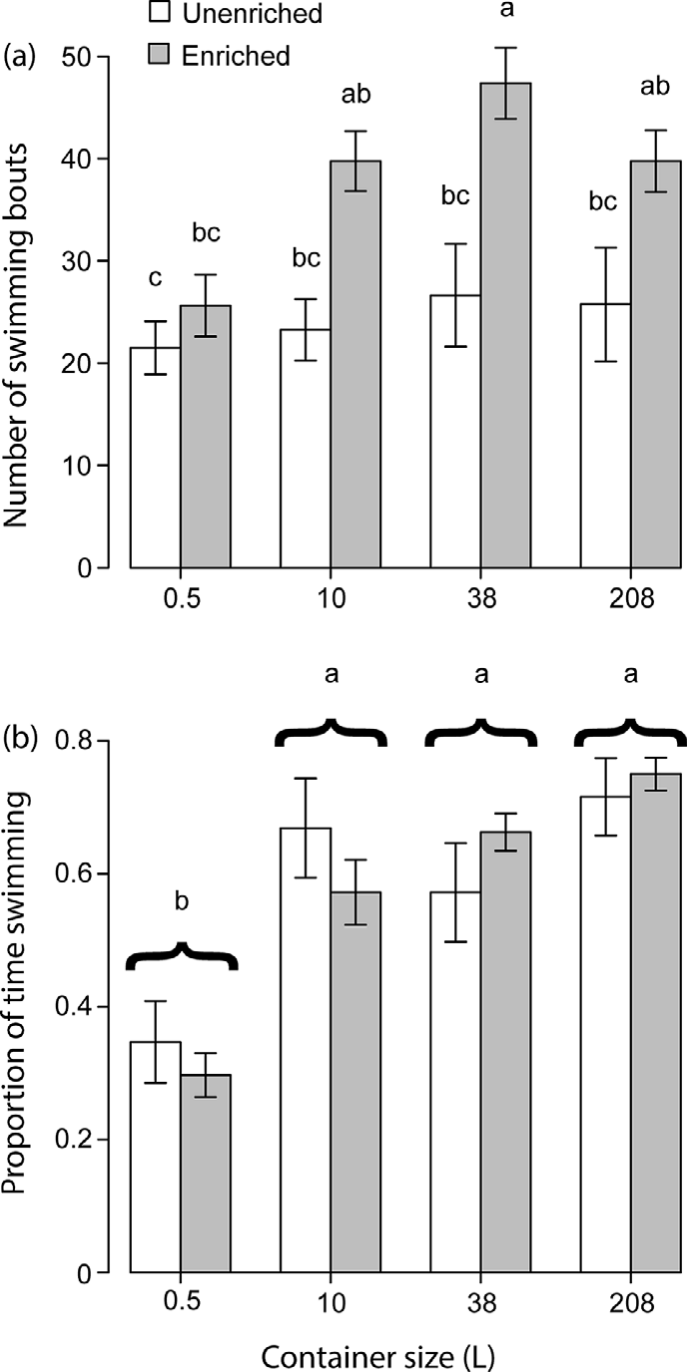
Showing (a) the mean number of swimming bouts performed by male *Betta splendens* in a 10-min period and (b) the proportion of time spent swimming in that same period. Means not sharing letters differ significantly according to Tukey HSD (*P* ≤ 0.021). Error bars show standard error.

The GLMM that analysed numbers of bouts of swimming found that swimming differed significantly across treatments (Corrected model: *F*_10,53_ = 3.672; *P* < 0.001). It did not find a significant effect of container size on swimming bouts (*F*_1,53_ = 1.337; *P* = 0.253) but it did find that enriched environments elicited more swimming bouts than did bare environments (*F*_1,53_ = 14.832; *P* < 0.001). The model found the ‘container size × enrichment’ interaction not to be significant (*F*_1,53_ = 0.003; *P* = 0.954). Different subjects were found not to exhibit different rates of swimming (*F*_7,53_ = 1.553; *P* = 0.170). ANOVA also found number of swimming bouts to be significantly higher in enriched containers overall (*F*_1,5_ = 27.707; *P* < 0.001), and the Tukey tests revealed several differences between unenriched and enriched treatments (Figure 2[a]).

The GLMM that analysed time budget found that amount of time spent swimming differed significantly across treatments (Corrected model: *F*_10,53_ = 7.886; *P* < 0.001). Different subjects were found to exhibit different proportions of time spent swimming (*F*_7,53_ = 4.744; *P* < 0.001). Even with this difference in behaviour across subjects, the model nevertheless found that behaviour also differed significantly across different container sizes (*F*_1,53_ = 27.134; *P* < 0.001), but not in bare vs enriched environments (*F*_1,53_ = 0.243; *P* = 0.624). The model also found the ‘container size × enrichment’ interaction not to be significant (*F*_1,53_ = 0.000; *P* = 0.990). Furthermore, the ANOVA found that container size had a significant effect on amount of time spent swimming overall (*F*_1,56_ = 21.392; *P* < 0.001), and Tukey tests revealed that fish spent significantly less time swimming in 0.5-L bowls than they did in the three larger aquaria (Figure 2[b]).

### Experiment 2

**Figure 3.**
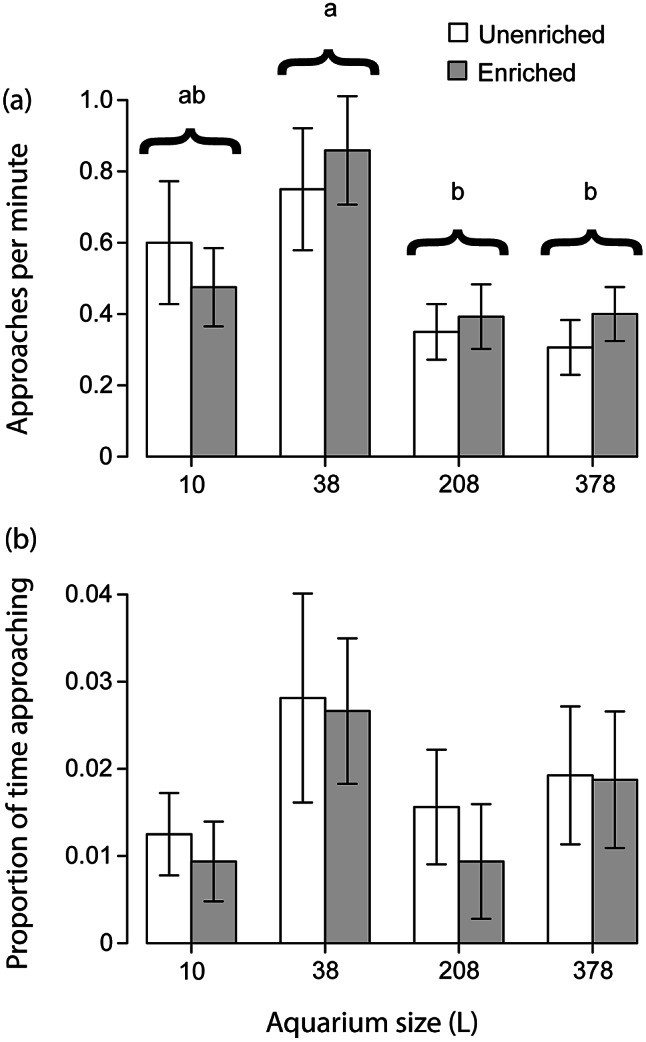
Showing (a) the mean frequency at which male *Betta splendens* approached another male held in the same aquarium and (b) the proportion of time male *B. splendens* spent approaching another male. Means not sharing letters differ significantly according to Tukey HSD (*P* ≤ 0.002). Error bars show standard error.

The statistical output from the GLMM and the ANOVAs used in Experiment 2 are shown in Table 2. The GLMM found that number of bouts of approach behaviour significantly differed across different subjects. The size of the aquarium also affected the number of approaches performed, as indicated by both the GLMM and the ANOVA, and the Tukey tests found that numbers of approaches were significantly lower in the two largest aquarium sizes compared to the 38-L aquaria (Figure 3[a]). The GLMM that tested time spent approaching other fish found an effect for subject, but no other factors affected time spent engaged in approach behaviour according to either the GLMM or the ANOVA (Figure 3[b]).

**Table 2.** Results of statistical analyses used in Experiment 2, which tested behaviour in diads of male *B. splendens* held in bare or enriched aquaria of various sizes

	GLMM				ANOVA			
	Bouts		Time budget		Bouts		Time budget	
Approaches	*F*	*P*	*F*	*P*	*F*	*P*	*F*	*P*
Corrected model	2.748	**<0.001**	1.915	**0.008**	–	–	–	–
Aquarium size	0.787	**0.002**	0.310	0.579	5.825	**0.002**	1.917	0.137
Enrichment	0.021	0.886	0.346	0.558	0.120	0.730	0.277	0.601
Aquarium size × Enrichment	0.624	0.432	0.012	0.914	0.383	0.766	0.054	0.983
Focal fish	3.582	**<0.001**	2.203	**0.011**	–	–	–	–
Opponent fish	0.904	0.562	1.618	0.083	–	–	–	–

	GLMM				ANOVA			
	Bouts		Time budget		Bouts		Time budget	
Displays	*F*	*P*	*F*	*P*	*F*	*P*	*F*	*P*
Corrected model	2.208	**0.002**	4.863	**<0.001**	–	–	–	–
Aquarium size	11.599	**<0.001**	2.406	0.124	4.480	**0.007**	3.295	**0.027**
Enrichment	3.207	0.077	13.864	**<0.001**	0.003	0.956	1.887	0.175
Aquarium size × Enrichment	0.491	0.485	2.506	0.117	0.947	0.424	2.538	0.066
Focal fish	2.919	**<0.001**	7.582	**<0.001**	–	–	–	–
Opponent fish	1.354	0.187	2.151	**0.014**	–	–	–	–

	GLMM				ANOVA			
	Bouts		Time budget		Bouts		Time budget	
Total attacks	*F*	*P*	*F*	*P*	*F*	*P*	*F*	*P*
Corrected model	2.836	**<0.001**	5.668	**<0.001**	–	–	–	–
Aquarium size	0.016	0.899	2.798	0.098	–	–	–	–
Enrichment	0.765	0.384	0.601	0.440	–	–	–	–
Aquarium size × Enrichment	0.022	0.881	2.790	0.098	–	–	–	–
Focal fish	3.202	**<0.001**	8.835	**<0.001**	–	–	–	–
Opponent fish	1.174	0.306*	1.173	0.306*	–	–	–	–

	GLMM				ANOVA			
	Bouts		Time budget		Bouts		Time budget	
Retreats	*F*	*P*	*F*	*P*	*F*	*P*	*F*	*P*
Corrected model	2.884	**<0.001**	2.481	**<0.001**	–	–	–	–
Aquarium size	4.757	**0.032**	0.220	0.640	1.452	0.237	1.292	0.286
Enrichment	1.460	0.230	0.972	0.327	0.169	0.683	0.250	0.619
Aquarium size × Enrichment	0.946	0.333	2.928	0.090	0.962	0.417	2.292	0.088
Focal fish	2.223	**0.010**	1.483	0.127	–	–	–	–
Opponent fish	3.293	**<0.001**	3.142	**<0.001**	–	–	–	–

	GLMM				ANOVA			
	Bouts		Time budget		Bouts		Time budget	
Gulp air	*F*	*P*	*F*	*P*	*F*	*P*	*F*	*P*
Corrected model	3.241	**<0.001**	1.358	0.128	–	–	–	–
Aquarium size	2.024	0.158	0.047	0.829	–	–	–	–
Enrichment	0.038	0.845	0.003	0.959	–	–	–	–
Aquarium size × Enrichment	2.857	0.094	0.073	0.787	–	–	–	–
Focal fish	4.510	**<0.001**	1.343	0.193	–	–	–	–
Opponent fish	1.457	0.138	1.642	0.077	–	–	–	–

	GLMM				ANOVA			
	Bouts		Time budget		Bouts		Time budget	
Forage	*F*	*P*	*F*	*P*	*F*	*P*	*F*	*P*
Corrected model	1.746	**0.019**	–	–	–	–	–	–
Aquarium size	10.643	**0.002**	–	–	1.653	0.188	–	–
Enrichment	1.689	0.197	–	–	0.074	0.786	–	–
Aquarium size × Enrichment	3.333	0.071	–	–	1.092	0.360	–	–
Focal fish	1.405	0.161	–	–	–	–	–	–
Opponent fish	1.302	0.217	–	–	–	–	–	–

	GLMM			
	Latency			
Latency to first bite	d.f. 1	d.f. 2	*F*	*P*
Corrected model	26	9	0.578	0.868
Aquarium size	1	9	0.639	0.445
Enrichment	1	9	0.376	0.555
Aquarium size × Enrichment	1	9	0.246	0.631
Biting fish	10	9	0.637	0.754
Opponent fish	13	9	0.412	0.929

GLMM corrected model d.f. = 33, 94; individual fish (focal or opponent) d.f. = 15, 94; each additional factor or interaction d.f. = 1, 94. ANOVA aquarium size d.f. = 3, 90; enrichment d.f. = 1, 90; interaction d.f. = 3, 90. *P*-values < 0.05 are highlighted in bold font.

* The repeated values here are not a mistake. It’s a coincidence.

**Figure 4.**
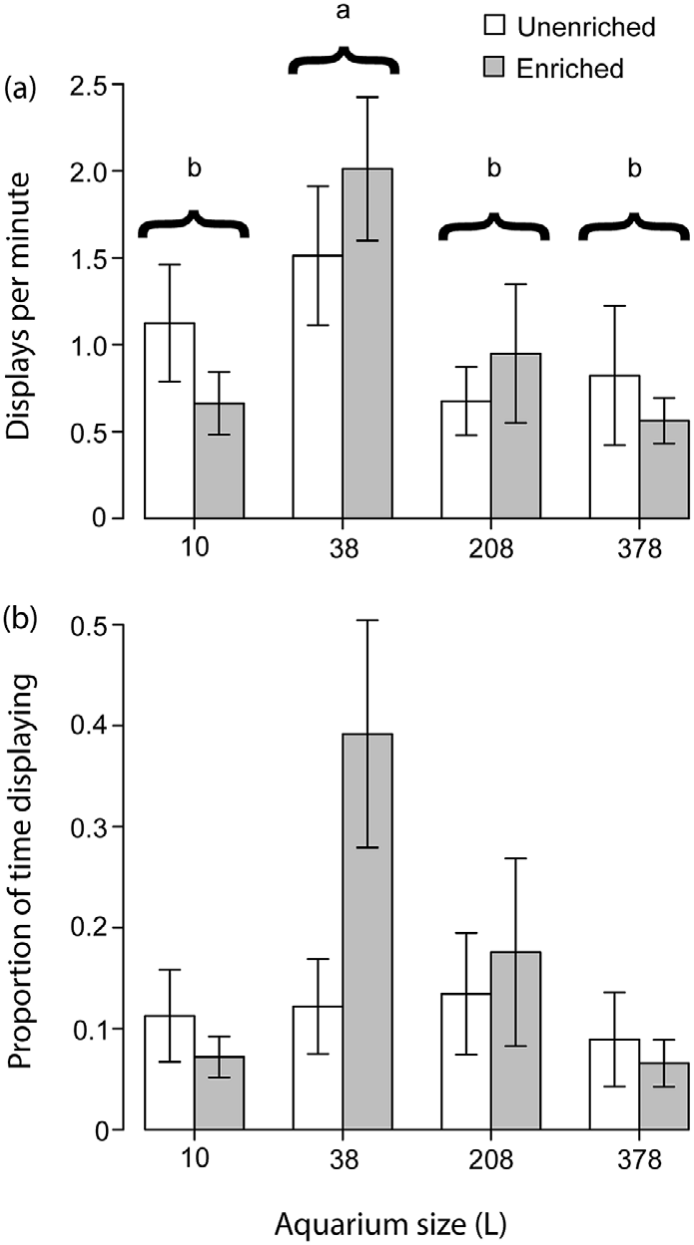
Showing (a) the mean frequency at which male *Betta splendens* aggressively displayed toward another male held in the same aquarium and (b) the proportion of time spent displaying. Means not sharing letters differ significantly according to Tukey HSD (*P* ≤ 0.045). Error bars show standard error.

The GLMM that analysed bouts of displays found that numbers of bouts differed across subjects. The size of the aquarium also affected the number of displays performed, according to both the GLMM and the ANOVA. Overall, fish performed fewer displays when in larger aquaria (Figure 4[a]). Displays were significantly higher in the 38-L aquaria than they were in the 208- and 378-L aquaria (as well as the 10-L aquaria). The GLMM that analysed time budget found that both the identity of the subject and the identity of the opponent fish affected amount of time that the subject spent displaying. The output also indicated that enrichment affected time spent displaying, but visual inspection of the data suggested that this significance was driven by outlying data in the 38-L enriched treatment (Figure 4[b]). It was not supported by the ANOVA, and we did not consider it further. The GLMM that analysed the combined variable ‘total attacks’ found a significant effect for focal fish, but not for any other factors. Even after combining three different attack patterns, the variable ‘total attack’ still occurred at low rates, and attack behaviour was not considered any further.

**Figure 5.**
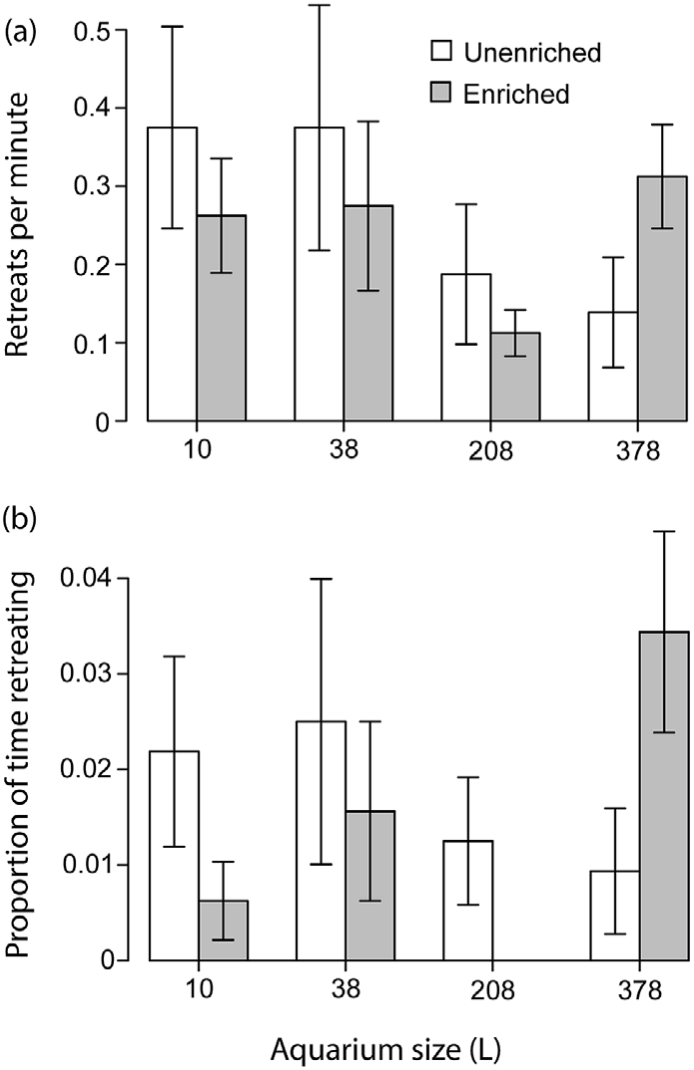
Showing (a) the mean frequency at which male *Betta splendens* retreated from another male held in the same aquarium (GLMM but not ANOVA found that aquarium size affected bouts of retreat behaviour, so no Tukey tests were performed; see text) and (b) the proportion of time spent retreating. Error bars show standard error.

Retreat behaviour mirrored display behaviour: The GLMM found that both the identity of the subject and aquarium size affected bouts of retreat behaviour (Figure 5[a]). In addition, the identity of the opponent fish also significantly affected the number of bouts of retreat behaviour performed by the subject. Just as individuals performed fewer displays in larger aquaria, they also performed fewer retreats in larger aquaria. The ANOVA did not find an effect of aquarium size on retreat behaviour, so no Tukey tests were performed. However, the significant effect of aquarium size indicated by the GLMM, and the pattern of fewer retreats in larger aquaria that is visible in the data, suggest that the inability of the ANOVA to detect an effect for aquarium size might be due to its inability to control for variation due to the identity of the subjects and the opponent fish. Therefore, we considered this effect to be authentic and we present the graph in Figure 5(a). Our time budget analysis found no significant effect of aquarium size, enrichment, or the identity of the subject (Figure 5[b]). However, identity of the opponent fish had a strong effect on time spent retreating.

**Figure 6.**
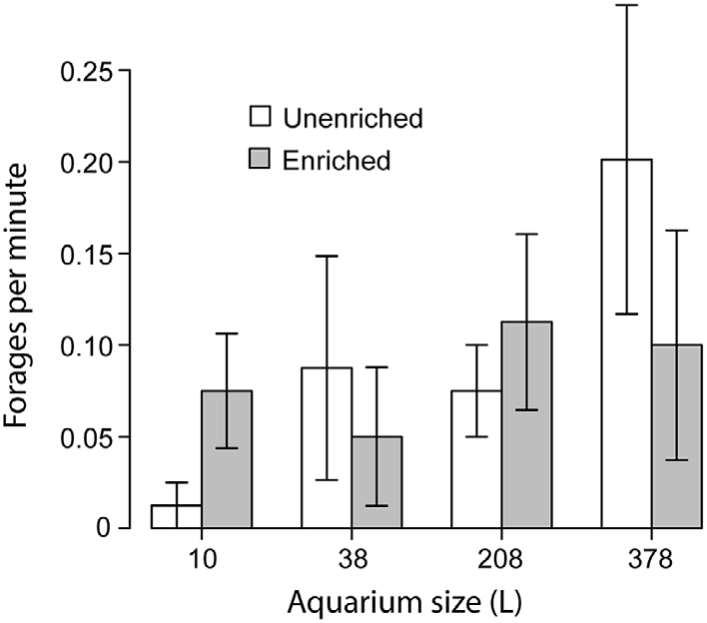
The mean frequency at which male *Betta splendens* performed bouts of foraging while another male was held in the same aquarium. GLMM but not ANOVA found that aquarium size affected bouts of foraging behaviour, so no Tukey tests were performed (see text). Error bars show standard error.

GLMM found a significant effect of aquarium size on bouts of foraging behaviour. The ANOVA was unable to detect this effect, so no Tukey tests were run. However, visual inspection of the data indicates this effect to be authentic: foraging was clearly more frequent in larger aquaria, so we present the graph as Figure 6. No other variable was found to affect foraging behaviour. Foraging behaviour was very rare in the time budget data (out of 128 fish-trials, there were 126 0-values and only two non-zero values), so we did not perform any analyses of time spent foraging. The GLMM found that different individuals performed significantly different rates of ‘gulping air’ behaviour. However, no other factors were found to affect gulping behaviour, so it was not analysed further. The GLMM that analysed latency to first bite found no significant effects.

## Discussion

This study found that confinement in small bowls reduces swimming behaviour in male *Betta splendens.* In Experiment 1, fish spent significantly less time swimming in small bowls than they did in the larger aquaria. *Bettas* are not strong swimmers, and they have been reported to spend time sheltering beneath rocks and plants (Pleeging & Moons 2017). In contrast, when our *Bettas* were placed in large aquaria they spent more than half of their time swimming. Swimming rates were similar across the 10-, 38-, and 208-L aquaria, suggesting that 10 L is sufficient to allow the full expression of swimming behaviour in *Bettas*, which corroborates advice commonly given to aquarium hobbyists (Betta Fish Care Guide 2021; Stanton 2021). However, other common advice is that bigger aquaria are always better. Although we did not formally quantify space use, we observed that fish swam throughout all the space provided to them, even in the largest aquaria.

Swimming has often been interpreted both as an indicator of wellness and as a beneficial, enjoyable experience in animals (e.g. Marshall *et al.* 2016), including fishes (Martins *et al.* 2012). In some contexts, locomotion can indicate poor welfare. In large mammalian carnivores, insufficient space may cause repetitive movement that is often interpreted as indicating poor welfare (Kroshko *et al.* 2016). In Atlantic halibut (*Hippoglossus hippoglossus*) grown in food fish aquaculture, swimming has been interpreted as poor welfare when not enough space was available to rest on the floor due to high stocking density (Kristiansen *et al.* 2004). However, in other contexts locomotion has been interpreted as a positive welfare indicator that might be inhibited by limited space or high densities (Oldfield 2011; Palstra & Planas 2011; Marshall *et al.* 2016). Opportunities for animals to roam, to forage, to hunt, to fight, to seek seclusion or the company of others, to take risks, and to make choices are widely acknowledged as necessary for positive well-being (Veasey 2017). The swimming behaviour observed in male *Bettas* in the current study appeared normal and not stereotypical or stress-related. Even if *Bettas* kept in small bowls are physically healthy, and they do not seem stressed, they are nevertheless prevented from fulfilling their capability to swim (Nussbaum 2006). We cannot know if our *Bettas* enjoyed the experience of swimming (Oldfield 2022). Determining what an animal is feeling has been called “the hardest biological problem of all” (Dawkins 2017). However, our data show that they will swim if given the opportunity to do so, and when confined in bowls their motivation to swim is not realised. Giving *Bettas* the benefit of the doubt would ensure that any motivation to swim that they might possess is not being frustrated (Birch 2017). Allowing *Bettas* to swim would also allow them to reap the physiological benefits of exercise (Palstra & Planas 2011), and it would make them more interesting for pet owners to observe.

Our results also found that *Bettas* performed more instances of swimming behaviour when they were in aquaria containing 3-D objects than when they were in bare bowls or aquaria. Physical structure is generally considered to provide enrichment for diverse vertebrate species (e.g. Scott & LaDue 2019), and it is often (but not always) beneficial for captive animals (Jones *et al.* 2021). In addition to providing cognitive stimulation, physical structure may also reduce stress by providing places to shelter (Näslund & Johnsson 2016), and given the choice, fish may prefer environments enriched with rocks and plants (Jones *et al.* 2021). In Experiment 1, *Bettas* in enriched treatments performed more swimming bouts than those in unenriched treatments. Since a new swimming bout was recorded every time the fish stopped swimming for at least one second, a higher number of swimming bouts indicates greater variation in behaviour. This suggests that physical enrichment may improve the welfare of *Bettas*, as behavioural diversity can be an indicator of positive welfare (Miller *et al.* 2020). Physical enrichment did not increase the number of swimming bouts in the 0.5-L bowls. This may be because the bowls were so small that the reduced amount of gravel and plants only negligibly enriched the environment, or because the added objects diminished the already limited space even further.

In Experiment 2, the numbers of times that fish approached and displayed to one another were lower in larger aquaria. This may suggest that greater available space prevented the fish from noticing one another, or that they were unwilling to expend energy to respond to distant stimuli. These results are consistent with those of Bronstein (1981), who found that increased inter-individual distance reduced aggression in physically separated male *Bettas.* The density provided in our largest treatments (2.4 individuals m^–2^) approached the 1.7 individuals m^–2^ density observed in territorial wild *Bettas* (Pleeging & Moons 2017), so we expected that our *Bettas* might cohabitate peacefully, but they did not. No effect of aquarium size on ‘total attack’ behaviour was noted. This opposes the results of Cain *et al.* (1980), who found that neither latency to first display nor number nor duration of displays varied with aquarium size, but that numbers of attacks were significantly lower in larger aquaria. The explanation for this apparent inconsistency is that Cain *et al.* (1980) defined “attack” as “a lunge and open-mouthed contact by the subject”. This behaviour is what we defined as a ‘bite’, and the occurrence of three consecutive bites resulted in us separating the fish and stopping recording data (also note that the range of aquarium sizes in Experiment 2 was much greater than the range used by Cain *et al*. 1980). We also found that latency to first bite was not affected by any factor. We expected that fish with more space and physical impediments would escalate to biting more slowly, and we expected more aggressive subjects to escalate faster. The fact that approaches and displays were lower in larger aquaria, but total attacks and latency to first bite were not, suggests that male *Bettas* might only be motivated to perform a low-intensity behaviour such as an approach when the distance is short and the metabolic cost of swimming is low, whereas *Bettas* sufficiently motivated to perform a high-intensity behaviour, such as an attack, will do so even if the cost is high.

Retreat behaviour mirrored display behaviour. Just as display behaviour was lower in larger aquaria, so was retreat behaviour. Furthermore, the identity of the opponent fish had a strong effect on retreat behaviour in the subject, as indicated by both number of bouts and time spent retreating. This is perhaps not surprising, because retreat behaviour should be expected to be a direct result of aggressive behaviour. In contrast, foraging behaviour was more frequent in larger aquaria. This might be due either to the fish perceiving more areas worth investigating for the presence of food, or due to less time being spent in proximity of the opponent fish so more time available to search for food.

Physical enrichment, on the other hand, did not reduce the amount of approach behaviour or agonistic behaviour performed. Presence of physical structure can decrease aggression through a variety of mechanisms. The most obvious being that opaque objects reduce the rate of visual contact between two individuals. Bronstein (1983) found male *Bettas* to be less aggressive when their view of a conspecific was partially obscured by plants, and that they possess only a brief within-fight memory for agonistic behaviour and the location of opponents (Bronstein 1989). Furthermore, in some fish species, an object can function as a landmark that the fish use as a territorial boundary, which encourages the formation of separate territories, thereby reducing aggression (Itzkowitz 1977; Breau & Grant 2002; Smith 2011). Finally, sufficient space and environmental complexity can make it uneconomical to behave aggressively. In juvenile Midas cichlids (*Amphilophus citrinellus*), Oldfield (2011) observed that when a sufficiently large “super complex” aquarium was provided, individuals seemed to ‘turn off’ aggressive behaviour and roam peacefully throughout the aquarium, often in close proximity to one another. Perhaps we did not include enough physical structure in our enriched treatments to elicit such a behavioural change in *Betta splendens.* Alternatively, domesticated *Bettas* might not ever ‘turn off’ aggressive behaviour. *Bettas* have been artificially selected for 1,000 years (Kwon *et al.* 2022), and perhaps there is some amount of sign stimuli that will stimulate aggressive behaviour regardless of ambient ecological conditions.

The identity of the subject had a significant effect on nearly every behaviour pattern we assessed, even behaviours that were not affected by any of the other factors we measured, such as gulping air. This is not surprising. Although *Bettas* have a reputation for behaving aggressively, aggression in *Bettas* is well known to vary greatly among individuals (Cain *et al.* 1980), so much so that they are often tested for aggressiveness before experiments so that non-aggressive individuals can be omitted (e.g. Bronstein 1983). Fortunately, the GLMMs we used were able to account for variation among individuals, so not only were we able to prevent individual variation from confounding our experiments, we were able to learn more about it.

### Animal welfare implications

The small fishbowls typically used in pet stores prevent male *Betta splendens* from performing swimming behaviour that they otherwise perform when sufficient space is provided. Male *Bettas* require physically enriched aquaria of least 10 L in volume to ensure full expression of swimming behaviour. Large, enriched spaces, however, are insufficient to ensure that multiple males will cohabitate peacefully. It seems unlikely that breeders, wholesalers, and retailers will follow this recommendation due to the higher costs required to house the fish (Saekhow *et al.* 2018; Thongprajukaew *et al.* 2023), but consumers might do so if their goal as pet owners is not to admire their *Bettas* as trophies but to nurture them to ensure that they thrive to the greatest extent possible.

## References

[r1] Balcombe J 2009 Animal pleasure and its moral significance. Applied Animal Behaviour Science 118(3-4): 208–216. 10.1016/j.applanim.2009.02.012

[r2] Betta Fish Care Guide 2021 *PetSmart.* https://www.petsmart.com/learning-center/fish-care/betta-fish-care-guide/A0188.html (accessed 7 December 2021).

[r3] Birch J 2017 Animal sentience and the precautionary principle. Animal Sentience 2: 1–15. 10.51291/2377-7478.1200

[r4] Boissy A, Manteuffel G, Jensen,MB, Moe RO, Spruijt B, Keeling LJ and Bakken M 2007 Assessment of positive emotions in animals to improve their welfare. Physiology and Behavior 92: 375–397. 10.1016/j.physbeh.2007.02.003 17428510

[r5] Brandao ML, Dorigão‐Guimarães F, Bolognesi MC, Gauy ACDS, Pereira AVS, Vian L and Gonçalves‐de‐Freitas E 2021 Understanding behaviour to improve the welfare of an ornamental fish. Journal of Fish Biology 99(3): 726–739. 10.1111/jfb.14802 34076258

[r6] Branson EJ 2008 Fish Welfare. John Wiley & Sons: London, UK. 10.1002/9780470697610

[r7] Breau C and Grant JWA 2002 Manipulating territory size via vegetation structure: Optimal size of area guarded by the convict cichlid (Pisces, Cichlidae). Canadian Journal of Zoology 80: 376–380. 10.1139/z02-002

[r8] Bronstein PM 1981 Social reinforcement in *Betta splendens*: A reconsideration. Journal of Comparative and Physiological Psychology 95: 943–950. 10.1037/h0077841 7195911

[r9] Bronstein PM 1983 Agonistic sequences and the assessment of opponents in male *Betta splendens*. The American Journal of Psychology 96(2): 163–177. 10.2307/1422809

[r10] Bronstein PM 1989 The priming and retention of agonistic motivation in male Siamese fighting fish, *Betta splendens*. Animal Behaviour 37(1): 165–166. 10.1016/0003-3472(89)90022-5

[r11] Cain NW, Jessen C and Flanagan M 1980 Social responsiveness and physical space as determinants of agonistic behavior in *Betta splendens*. Animal Learning & Behavior 8(3): 497–501. 10.3758/BF03199640

[r12] Dawkins MS 2017 Animal welfare with and without consciousness. Journal of Zoology 301(1): 1–10. 10.1111/jzo.12434

[r13] Fife-Cook I and Franks B 2019 Positive welfare for fishes: Rationale and areas for future study. Fishes 4(2): 31. 10.3390/fishes4020031

[r14] Goldstein SR 1975 Observations on the establishment of a stable community of adult male and female Siamese fighting fish (*Betta splendens*). Animal Behaviour 23: 179–185. 10.1016/0003-3472(75)90063-9

[r15] Haller J 1994 Biochemical costs of a three day long cohabitation in dominant and submissive male *Betta splendens*. Aggressive Behavior 20: 369–378. 10.1002/1098-2337(1994)20:5<369::AID-AB2480200504>3.0.CO;2-F

[r16] Haller J and Wittenberger C 1988 Biochemical energetics of hierarchy formation in *Betta splendens*. Physiology & Behavior 43*:* 447–450. 10.1016/0031-9384(88)90118-7 3194464

[r17] Huntingford FA, Adams C, Braithwaite VA, Kadri S, Pottingers TG, Sandoe P and Turnbull JF 2006 Current issues in fish welfare. Journal of Fish Biology 68: 332–372. 10.1111/j.0022-1112.2006.001046.x

[r18] Itzkowitz M 1977 Interrelationships of dominance and territorial behavior in the pupfish *Cyprinodon variegatus*. Behavioural Processes 2: 383–391. 10.1016/0376-6357(77)90008-0 24896902

[r19] Jones NAR, Webster MM and Salvanes AGV 2021 Physical enrichment research for captive fish: Time to focus on the DETAILS. Journal of Fish Biology 99(3): 704–725. 10.1111/jfb.14773. 33942889

[r20] Kristiansen TS, Fernö A, Holm JC, Privitera L, Bakke S and Fosseidengen JE 2004 Swimming behaviour as an indicator of low growth rate and impaired welfare in Atlantic halibut (*Hippoglossus hippoglossus L.*) reared at three stocking densities. Aquaculture 230: 137–151. 10.1016/S0044-8486(03)00436-8

[r21] Kristiansen TS and Bracke MB 2020 A brief look into the origins of fish welfare science. In Kristiansen et al. (eds). The Welfare of Fish. pp. 1–17. London, UK: Springer. 10.1007/978-3-030-41675-1_1

[r22] Kristiansen TS, Fernö A, Pavlidis MA and van de Vis H 2020 The Welfare of Fish. Springer: London, UK. 10.1007/978-3-030-41675-1

[r23] Kroshko J, Clubb R, Harper L, Mellor E, Moehrenschlager A and Mason G 2016 Stereotypic route tracing in captive Carnivora is predicted by species-typical home range sizes and hunting styles. Animal Behaviour 117: 197–209. 10.1016/j.anbehav.2016.05.010

[r24] Kwon YM, Vranken N, Hoge C, Lichak MR, Norovich AL, Francis KX and Bendesky A 2022 Genomic consequences of domestication of the Siamese fighting fish. Science Advances 8(10): eabm4950. 10.1126/sciadv.abm4950 PMC8906746 35263139

[r25] Marshall AR, Deere NJ, Little HA, Snipp R, Goulder J and Mayer-Clarke S 2016 Husbandry and enclosure influences on penguin behavior and conservation breeding. Zoo Biology 35: 385–397. 10.1002/zoo.21313 27486862

[r26] Martin P and Bateson P 2007 Measuring Behavior: An Introductory Guide, Third Edition. Cambridge University Press: Cambridge, UK.

[r27] Martins CIM, Galhardo L, Noble C, Damsgård B, Spedicato MT, Zupa W, Beauchaud M, Kulczykowska E, Massabuau J, Carter T, Planellas SR and Kristiansen T 2012 Behavioural indicators of welfare in farmed fish. Fish Physiology and Biochemistry 38: 17–41. 10.1007/s10695-011-9518-8 21796377 PMC3276765

[r28] Miller LJ, Vicino GA, Sheftel J and Lauderdale LK 2020 Behavioral diversity as a potential indicator of positive animal welfare. Animals 10: 1211. 10.3390/ani10071211 32708625 PMC7401597

[r29] Näslund J and Johnsson JI 2016 Environmental enrichment for fish in captive environments: effects of physical structures and substrates. Fish and Fisheries 17: 1–30. 10.1111/faf.12088

[r30] Nussbaum MC 2006 The moral status of animals. Chronicle of Higher Education 52(22): B6–8. 16789292

[r31] Oldfield RG 2011 Aggression and welfare in a common aquarium fish, the Midas cichlid. Journal of Applied Animal Welfare Science 14: 340–360. 10.1080/10888705.2011.600664 21932947

[r32] Oldfield RG 2022 You can’t betray a fish: One reason eating fish may cause less harm than eating cows. Journal of Animal Ethics 12: 51–58. 10.5406/21601267.12.1.05

[r33] Oldfield RG and Bonano P 2023 Psychological and social well-being of bony fishes in zoos and aquariums. Zoo Biology 42: 185–193. 10.1002/zoo.21729 36065963

[r34] Palstra AP and Planas JV 2011 Fish under exercise. Fish Physiology and Biochemistry 37: 259–272. 10.1007/s10695-011-9505-0 21611721 PMC3107430

[r35] PETA 2021 *PETA Asia reveals horrors in the global betta fish trade.* PETA Exposés and Undercover Investigations. https://investigations.peta.org/petco-betta-fish-supplier-thailand/ (accessed 22 November 2021)

[r36] Pleeging CCF and Moons CPH 2017 Potential welfare issues of the Siamese fighting fish (*Betta splendens*) at the retailer and in the hobbyist aquarium. Vlaams Diergeneeskundig Tijdschrift 86: 213–223. 10.21825/vdt.v86i4.16182

[r37] Saekhow S, Thongprajukaew K, Phromkunthong W and Sae-Khoo H 2018 Minimal water volume for intensively producing male Siamese fighting fish (*Betta splendens* Regan, 1910). Fish Physiology and Biochemistry 44: 1075–1085. 10.1007/s10695-018-0495-z 29603077

[r38] Saxby A, Adams L, Snellgrove D, Wilson RW and Sloman KA 2010 The effect of group size on the behaviour and welfare of four fish species commonly kept in home aquaria. Applied Animal Behaviour Science 125(3-4): 195–205. 10.1016/j.applanim.2010.04.008

[r39] Scott NL and LaDue CA 2019 The behavioral effects of exhibit size versus complexity in African elephants: A potential solution for smaller spaces. Zoo Biology 38(5): 448–457. 10.1002/zoo.21506 31271671

[r40] Sloman KA, Baldwin L, McMahon S and Snellgrove D 2011 The effects of mixed-species assemblage on the behaviour and welfare of fish held in home aquaria. Applied Animal Behaviour Science 135(1-2): 160–168. 10.1016/j.applanim.2011.08.008

[r41] Smith C 2011 Good fences make good neighbours: The role of landmarks in territory partitioning in the rose bitterling (*Rhodeus ocellatus*). Behaviour 148(2): 233–246. 10.1163/000579511X554233

[r42] Smith A and Gray H 2011 Goldfish in a tank: the effect of substrate on foraging behaviour in aquarium fish. Animal Welfare 20: 311–319. 10.1017/S0962728600002876

[r43] Stanton L 2021 *How much space do* Betta *fish need? A Betta fish tank guide! It’s a fish thing.* https://www.itsafishthing.com/how-much-space-do-betta-fish-need/ (accessed 7 December 2021)

[r44] Stevens CH, Croft DP, Paull GC and Tyler CR 2017 Stress and welfare in ornamental fishes: what can be learned from aquaculture? Journal of Fish Biology 91(2): 409–428. 10.1111/jfb.13377 28691234

[r45] The Herald 2009 *A fine catch of outrage thanks to piscatorial petitioners.* The Herald. https://www.heraldscotland.com/default_content/12769270.fine-catch-outrage-thanks-piscatorial-petitioners/ (accessed 20 August 2023)

[r46] Thongprajukaew K, Takaeh S, Esor N, Saekhow S, Malawa S, Nuntapong N and Choodum A 2023 Optimal water volume for transportation of male Siamese fighting fish (*Betta splendens*). Aquaculture Reports 28: 101430. 10.1016/j.aqrep.2022.101430

[r47] Torgersen T 2020 The Welfare of Fish. Springer: London, UK. 10.1007/978-3-030-41675-1_15

[r48] Veasey JS 2017 In pursuit of peak animal welfare; the need to prioritize the meaningful over the measurable. Zoo Biology 36: 413–425. 10.1002/zoo.21390 29193216

[r49] Walster C, Rasidi E, Saint-Erne N and Loh R 2015 The welfare of ornamental fish in the home aquarium. Companion Animal 20(5): 302–306. 10.12968/coan.2015.20.5.302

[r50] Watters JV, Krebs BL and Eschmann CL 2021 Assessing animal welfare with behavior: Onward with caution. Journal of Zoological and Botanical Gardens 2: 75–87. 10.3390/jzbg2010006

